# Inflammatory-Dependent Bidirectional Effect of Bile Acids on NLRP3 Inflammasome and Its Role in Ameliorating CPT-11-Induced Colitis

**DOI:** 10.3389/fphar.2022.677738

**Published:** 2022-05-31

**Authors:** Chuyao Liao, Di Wang, Siyuan Qin, Ying Zhang, Jie Chen, Ruijie Xu, Fengguo Xu, Pei Zhang

**Affiliations:** Key Laboratory of Drug Quality Control and Pharmacovigilance (Ministry of Education), State Key Laboratory of Natural Medicine, China Pharmaceutical University, Nanjing, China

**Keywords:** bile acid, NLRP3 inflammasome, Sp1, tgr5, CPT-11-induced colitis

## Abstract

Irinotecan (CPT-11) in combination with 5-fluorouracil and leucovorin is a first-line chemotherapy regimen for the treatment of colorectal cancer; however, its clinical application is limited by the dose-limiting gastrointestinal toxicity of colitis. In our previous studies, several bile acids (BAs) were found significantly elevated in the colon of the CPT-11-induced rat colitis model. On the other hand, NLRP3 inflammasome has been reported to play important roles in mediating colitis. Interestingly, BA was stated to activate the NLRP3 inflammasome in some studies, while in some other reports, it showed an inhibitory effect. We assumed that the inflammatory status in different circumstances might have contributed to the controversial findings. In this study, we first discovered, under non-inflammatory conditions, that supplementing BA could activate the NLRP3 inflammasome in THP-1-differentiated macrophages and promote inflammation. In lipopolysaccharide (LPS)-induced inflammatory macrophages, however, BA inhibited the NLRP3 inflammasome and reduced inflammation. Further experiments demonstrated that Takeda G protein-coupled receptor 5 (TGR5) is essential in mediating the inhibitory effect of BA, while phospho-SP1 (p-SP1) is key to the activation. Furthermore, we applied the above findings to ameliorate CPT-11-caused colitis in rats by inhibiting SP1 with mithramycin A (MitA) or activating TGR5 using oleanolic acid (OA). Our findings may shed light on the discovery of effective interventions for reducing dose-limiting chemotherapy-induced colitis.

## Graphical Abstract

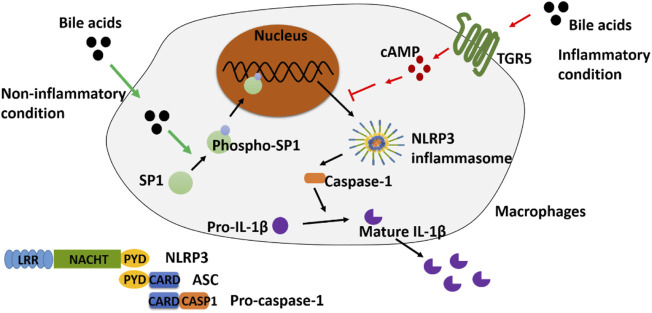

## Introduction

Bile acids (BAs) are hydroxylated steroids, synthesized from cholesterol in the liver. They play important roles in regulating lipid, glucose, and energy metabolism (McGlone and Bloom, 2019). Disorders in BA homeostasis are associated with cholestatic liver diseases, dyslipidemia, fatty liver diseases, cardiovascular diseases, and diabetes (Chiang, 2013). BA dysregulation is also found closely related to intestinal diseases, such as inflammatory bowel disease and diarrhea (Vítek, 2015; Vijayvargiya and Camilleri, 2019; Sinha et al., 2020). In colitis, the metabolic disorder of BA is an important risk factor for inflammation. These effects of BA are mostly accomplished by simulating its receptors such as BA-activated receptors, especially Farnesoid X receptor (FXR), TGR5, and sphingosine-1-phosphate receptor 2 (S1PR2) (Biagioli and Carino, 2017; Hou et al., 2018; Zhao et al., 2018). For example, it was reported that BA can exacerbate colitis by up-regulating S1PR2 in mice (Zhao et al., 2018). However, there are also studies showing that the activation of TGR5 and FXR can lead to an anti-inflammatory effect (Chávez-Talavera et al., 2017; Hou et al., 2018).

In the interaction between BA and inflammation, the role of NLRP3 inflammasome is widely recognized. However, there are controversies about the effect of BA on the NLRP3 inflammasome. Many studies claimed that BA could activate NLRP3 inflammasome (Gong et al., 2016; Zhao et al., 2016; Hao et al., 2017), while others concluded that BA had an inhibitory effect on NLRP3 inflammasome (Guo et al., 2016a). For example, Wang et al. reported that most BA including cholic acid (CA), glycocholic acid (GCA), chenodeoxycholic acid (CDCA), deoxycholic acid (DCA), ursodeoxycholic acid (UDCA), lithocholic acid (LCA), and taurolithocholic acid (TLCA) could significantly inhibit nigericin-induced NLRP3 inflammasome activation and IL-1β production in macrophages via the TGR5–cAMP–PKA axis (Guo et al., 2016a), while Gonzalez et al. demonstrated that CDCA and DCA promoted NLRP3 inflammasome activation and IL-1β production in various types of macrophages (Hao et al., 2017). The key factors contributing to the opposite effect of BA on NLRP3 inflammasome as well as the underlying mechanism remain elusive.

CPT-11, known as a chemotherapeutic agent, is a selective inhibitor of DNA topoisomerase I. The combination of CPT-11 with 5-fluorouracil and leucovorin is the first-line chemotherapy for the treatment of metastatic colorectal cancer (Sears et al., 1999; Sandmeier et al., 2005). However, CPT-11 could cause severe gastrointestinal toxicity including colitis, which greatly limited its clinical use (Sandmeier et al., 2005; Wang et al., 2020). In our previous metabolomics studies, we found that the metabolism of BA was disturbed in CPT-11-induced colitis in rats, manifested by the significant up-regulated levels of CDCA, DCA, GDCA, and TDCA in the colon tissue (Wang et al., 2015). On the other hand, recent studies indicate that NLRP3 inflammasome plays an essential role in colitis induced by CPT-11, and there is evidence showing that CPT-11 could activate NLRP3 inflammasome and cause inflammation both *in vitro* and *in vivo* (Li et al., 2015; Huang et al., 2020).

In the current study, in light of the vital role of BA in colitis, we investigated the potential mechanism underlying the aforementioned conflicting effect of BA on the NLRP3 inflammasome. As studies have reported, the release of inflammatory factors (for example, TNF-α, IL-6, and IL-1β) can be extensively promoted by LPS stimulation in phorbol 12-myristate 13-acetate (PMA)-differentiated THP-1 cells (Zou et al., 2017; Zhao D. et al., 2019). In the current study, “inflammatory condition” or “non-inflammatory condition” was defined to distinguish the state of THP-1-induced macrophages that receive LPS stimulation or not, respectively. We found that BA could activate NLRP3 inflammasome via promoting p-SP1 under non-inflammatory conditions, while under inflammatory conditions, BA promoted the expression of TGR5 and led to the inhibition of the NLRP3 inflammasome *in vitro*. Utilizing these findings, *in vivo* experiments were designed, and the results showed that the colitis caused by CPT-11 was remarkably ameliorated with the inhibition of SP1 or activation of TGR5. Taken together, our findings may assist in discovering effective interventions for reducing chemotherapy-induced colitis.

## Materials and Methods

### Chemicals and Reagents

DCA, CDCA, GDCA, and PMA were purchased from Sigma-Aldrich (St. Louis, MO, United States). TDCA was purchased from J&K (Manhattan, NY, United States). MitA and SBI-115 were purchased from MedChemExpress (Monmouth Junction, NJ, United States). OA was purchased from Aladdin ^®^ (Los Angeles, CA, United States). Roswell Park Memorial Institute (RPMI) 1640 medium and fetal bovine serum were purchased from Gibco (Grand Island, NY, United States). HEPES buffer was purchased from Boster (Wuhan, China). Anti-NLRP3 (Lot#: 19771-1-AP), anti-caspase-1/p20/p10 (#:22915-1-AP), anti-SP1 (Lot#: 21962-1-AP), anti-β-actin (Lot#: 66009-1-Ig), and HRP-conjugated beta actin monoclonal antibody (Lot#: HRP-60008) were obtained from Proteintech (Chicago, IL, United States). Anti-p-SP1 (Lot#: AF3121) was obtained from Affinity (Affinity Biosciences, United States). Anti-GPBAR1 (Lot#: BS60582) was purchased from Bioworld Technology (MN, United States). Anti-Pro-IL-1β (Lot#: WL02257) and anti-mature-IL-1β (Lot#: WL00891) were purchased from Wanleibio (Shenyang, China). Radioimmunoprecipitation (RIPA) buffer, bicinchoninic acid (BCA) protein assay kit, and loading buffer were purchased from Beyotime Biotechnology (Shanghai, China). Phenylmethylsulfonyl fluoride (PMSF) was purchased from Thermo Fisher Scientific (Waltham, MA, United States). RNAiso Plus and PrimeScript™ RT reagent Kit were purchased from TaKaRa (TaKaRa Biotechnology, Dalian, China). IL-1β, IL-6, and TNF-α enzyme-linked immunosorbent assay (ELISA) kits were purchased from 4A Biotech (Co., Ltd., Beijing, China). ELISA kit for the measurement of cyclic AMP (cAMP) was purchased from GenScript (Nanjing, China).

### Cell Culture

THP-1 cells were cultured in RPMI 1640 medium supplemented with 10% fetal bovine serum, 1× HEPES buffer, 100 U/ml of penicillin, and 100 μg/ml of streptomycin. THP-1 monocytes were differentiated into macrophages by stimulating with 100 ng/ml of PMA for 48 h. The cells were then cultured in a serum-free medium for 24 h to enhance the differentiation.

### Cell Viability Assay

THP-1 monocytes were seeded into 96-well plates at a density of 3 × 10^5^ cells/well. After differentiation, cells were exposed to BA or cell culture medium as vehicle for 48 h. Cell viability was measured by the 3-(4,5-dimethylthiazol-2-yl)-2,5-diphenyl-2H-tetrazolium bromide (MTT) assay.

### Animal Experiments and Sample Collection

Fifty healthy 6–8-week-old male specific-pathogen-free Sprague-Dawley rats weighing 180–200 g were purchased from Vital River Laboratory Animal Technology Co., Ltd. (Pinghu, China, Permission No. SCXK (Zhe) 2019-0001). The animals were housed in a temperature-controlled environment (24 ± 2°C) with a standard rodent diet under a 12 h/12 h-dark/light cycle. All animal studies and procedures were conducted in accordance with the United States National Institutes of Health Guide for the Care and Use of Laboratory Animals and approved by the Animal Ethics Committee of China Pharmaceutical University (License No. SYXK 2018-0019).

After a week of acclimatization, the animals were randomly divided into five groups (*n* = 10) including the control, model, MitA, OA, and MitA+OA groups. The detailed procedure of the animal experiment can be found in Supplementary Figure S1. Briefly, the individuals in the MitA+OA group were injected with MitA (intraperitoneally, 0.15 mg/kg) (Wei et al., 2016) and OA (intragastrically, 100 mg/kg) for five consecutive days from day 1 and CPT-11 (intravenously, 120 mg/kg) each day for two consecutive days from day 2. For the MitA group, the administration was similar to that of the MitA+OA group except that 0.5% CMC-Na (solvent of OA) was given instead of OA. Similarly, normal saline (solvent of MitA) was given to the OA individuals instead of MitA and the rest was in accordance with the MitA+OA group. Individuals in the model group were receiving equivalent 0.5% CMC-Na and normal saline, and CPT-11. In addition, 0.5% CMC-Na, normal saline and the solvent of CPT-11 (Trifan et al., 2002; Mego et al., 2015) were administered to the individuals in the control group as vehicle.

The diarrhea score of each animal was monitored twice a day referring to the scoring criteria in the existing literature (Kurita et al., 2000). The colon tissue was collected on day 6. After being drained of feces, the colon tissue was washed with normal saline, and then a portion of the proximal colon of each rat was fixed in 10% formalin for histological examination and the rest (middle and distal) were stored at −80°C for Western blotting and ELISA analysis.

### Enzyme-Linked Immunosorbent (ELISA) Assay

The contents of cAMP, IL-1β, IL-6, and TNF-α in cell culture supernatants or colon tissue homogenates were quantified by ELISA kits according to the manufacturer’s instructions.

### Western Blotting

Mature-IL-1β, pro-IL-1β, caspase-1, NLRP3, SP1, p-SP1, TGR5 (GPBAR1), and β-actin expression were analyzed using standard Western blotting protocols. Cells and tissues were lysed by RIPA buffer containing 1 mmol/L of PMSF, and total proteins were extracted according to the manufacturer’s protocols. Then, the protein concentration was measured using the BCA protein assay kit. Proteins (30 μg) were separated by SDS-polyacrylamide and transferred to polyvinylidene difluoride membranes (0.2 μm, Millipore, MA, United States). The membranes were blocked with 5% (w/v) nonfat milk for 2 h at room temperature and incubated with primary antibodies at 4°C overnight. After being washed three times with PBST, the membranes were incubated with secondary antibodies conjugated to horseradish peroxidase for approximately 1.5 h at room temperature. Then, the immunoreactive bands were visualized using enhanced chemiluminescence (ECL) (Millipore) by a Tanon 5200 chemiluminescent imaging system (Tanon Science and Technology). The relative protein expression was calculated by densitometric analysis using ImageJ software.

### mRNA Preparation and qRT-PCR

Total RNA was extracted from THP-1 monocytes using RNAiso Plus Kit. Then, the RNA concentration was measured by a Nano-Drop 2000 (Thermo Fisher Scientific, Waltham, MA, United States). Complementary DNA (cDNA) was obtained by reverse transcription with the PrimeScript™ RT Reagent Kit. Subsequently, qRT-PCR was performed using SYBR Green I Master (Roche Diagnostics, Basel, Switzerland) on a LightCycler 480 (Roche) following the manufacturer’s instructions. The sequences of the PCR primers used are as follows: IL-1β forward 5′-ATG​ATG​GCT​TAT​TAC​AGT​GGC​AA-3′ and reverse 5′-GTC​GGA​GAT​TCG​TAG​CTG​GA-3′; NLRP3 forward 5′-CGT​GAG​TCC​CAT​TAA​GAT​GGA​GT-3′ and reverse 5′-CCC​GAC​AGT​GGA​TAT​AGA​ACA​GA-3′’; and β-actin forward 5′-ATT​GCC​GAC​AGG​ATG​CAG​AA and reverse 5′-GCT​GAT​CCA​CAT​CTG​CTG​GAA-3′. Results were normalized to the internal control β-actin, and the expression was calculated by the 2^−△△CT^ method (Khan-Malek and Wang, 2017).

### Statistical Analysis

Statistical analysis was performed using GraphPad Prism 5.0 software (GraphPad Software Inc., La Jolla, CA, United States). All *in vitro* experiments were repeated at least three times independently with at least three replicates, and the results were presented as mean ± standard deviation (SD) unless otherwise specified. Independent unpaired two-tailed Student’s *t*-test was performed to evaluate the differences between two groups, and one-way analysis of variance with Bonferroni correction was performed for multiple comparisons. *p* < 0.05 was considered statistically significant. The survival rate was summarized by Kaplan–Meier survival curves.

## Results

### BAs Activate NLRP3 Inflammasome in Non-Inflammatory Conditions

**FIGURE 1:**
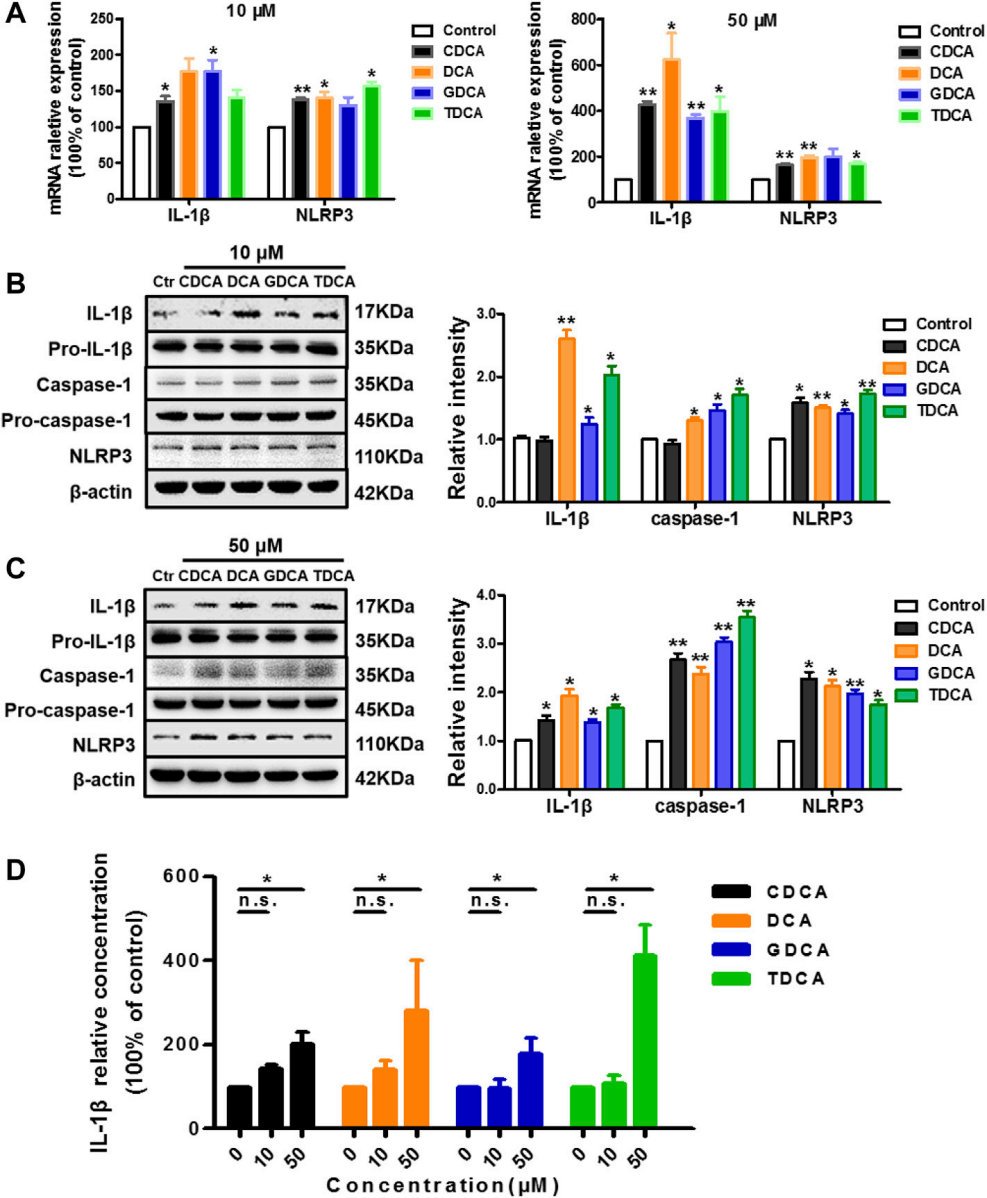
BAs activate NLRP3 inflammasome in non-inflammatory conditions in macrophages. **(A)** Relative mRNA expression of NLRP3 and IL-1β with the treatment of BA at 10 or 50 μM. IL-1β, caspase-1, and NLRP3 protein levels with the treatment of BA at **(B)** 10 μM and **(C)** 50 μM. **(D)** ELISA analysis of IL-1β in culture medium after treating with BAs at 10 and 50 μM. Data are presented as mean ± SD (*n* = 3). Statistical analysis was performed using Student’s *t* test (*p < 0.05, **p < 0.01, and n.s., not significant).

To assess the effects of the four BAs on NLRP3 inflammasome in non-inflammatory conditions, we treated macrophages differentiated from THP-1 monocytes for 4 h with 10 and 50 μM of CDCA, DCA, GDCA, or TDCA. The concentrations were selected according to the published data (Guo et al., 2016a; Hao et al., 2017) and our cell viability assay results (Supplementary Figure S2A). At a concentration of 10 μM, CDCA and GDCA activated the mRNA expression of IL-1β; however, DCA, GDCA, and TDCA significantly activated the expression of IL-1β and caspase-1 in protein, while CDCA hardly showed activating effects on it (Figures 1A,B). When the concentration was increased to 50 μM, all BAs remarkably increased the mRNA and protein expression of IL-1β, as well as caspase-1 protein expression (Figures 1A,C). Little impact on pro-IL-1β and pro-caspase-1 was observed (Figures 1B,C). Besides, all BAs activated NLRP3 protein expression at both 10 and 50 μM (Figures 1B,C), although GDCA failed to present a significant promoting effect on the mRNA expression of NLRP3 (Figure 1A). Moreover, the IL-1β level in the culture medium also increased with the BA treatment, especially at 50 μM (Figure 1D). These results suggested that BA can activate the NLRP3 inflammasome and show a pro-inflammatory effect in non-inflammatory conditions.

### BAs Inhibit NLRP3 Inflammasome in Inflammatory Conditions

**FIGURE 2:**
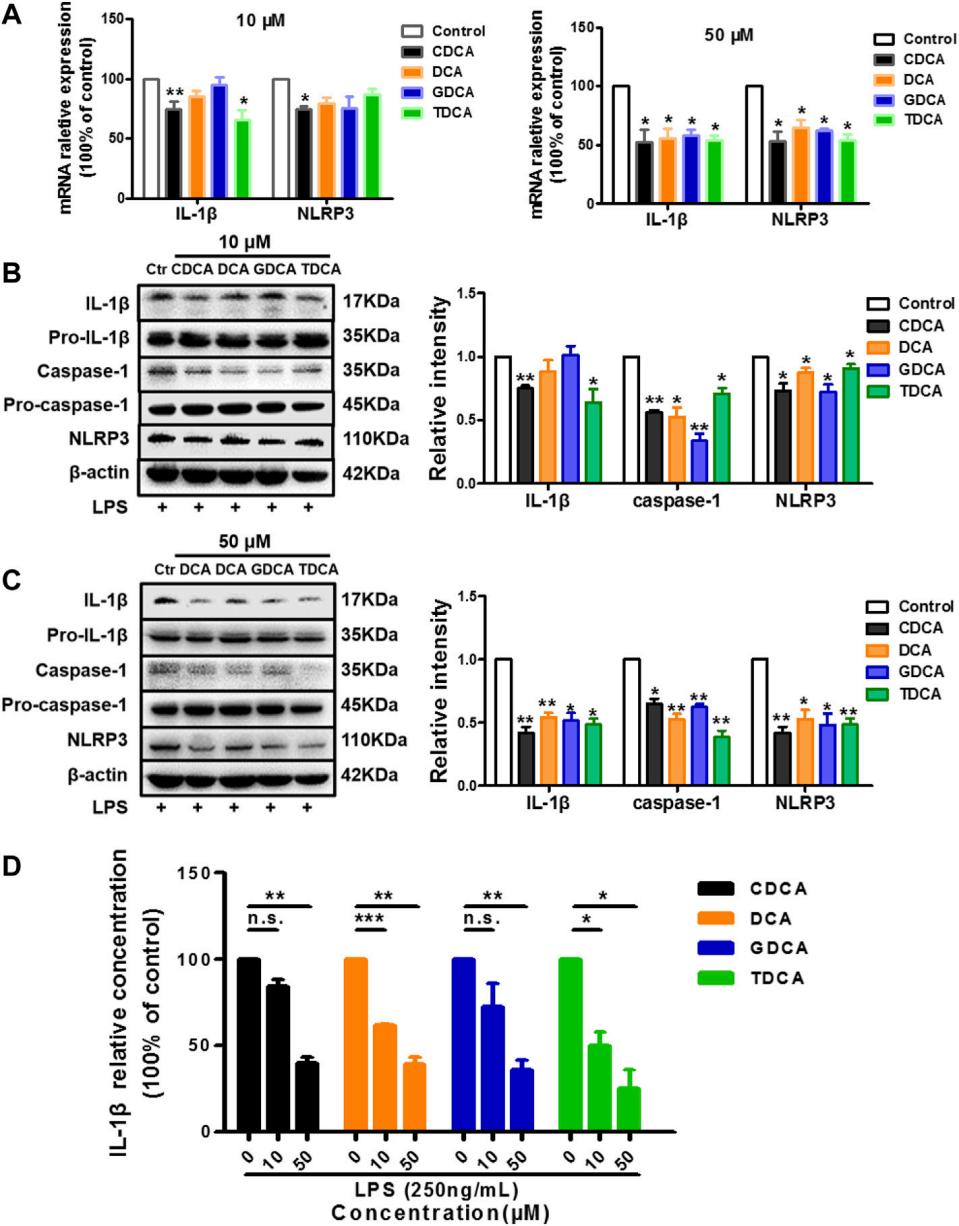
BAs inhibit NLRP3 inflammasome in inflammatory conditions in macrophages. Prior to the BA treatment, cells were pretreated with 250 ng/ml LPS for 1 h. **(A)** Relative mRNA expression of NLRP3 and IL-1β with BAs at 10 or 50 μM. IL-1β, caspase-1, and NLRP3 protein levels after treating with BAs at 10 μM **(B)** and **(C)** 50 μM. **(D)** IL-1β in culture medium after treating with BAs at 10 and 50 μM. Data are presented as mean ± SD (*n* = 3). Statistical analysis was performed using Student’s *t* test (*p < 0.05, **p < 0.01, ***p < 0.001, and n.s., not significant).

As reported in the existing literature, LPS incubation with macrophages differentiated from THP-1 monocytes could lead to a sharp increase in inflammatory factors (Kuijk et al., 2008; Zhao D. et al., 2019; Zhao W. et al., 2019). Our pre-experiments also confirmed this (data not shown). Therefore, to establish an inflammatory condition, we pretreated the macrophages differentiated from THP-1 monocytes with 250 ng/ml of LPS for 1 h before BA stimulation. Opposite to what we observed in non-inflammatory conditions, 10 μM of CDCA and TDCA inhibited IL-1β on mRNA and protein level (Figures 2A,B). Although only CDCA showed a significant inhibitory effect on the NLRP3 mRNA expression, all the BAs significantly inhibited NLRP3 protein at 10 μM (Figures 2A,B). At a concentration of 50 μM, all BAs could significantly inhibit the mRNA and protein expressions of IL-1β and NLRP3 (Figures 2A,C). Besides, pro-IL-1β and pro-caspase-1 were barely affected, while caspase-1 was inhibited by BA at both 10 and 50 μM (Figures 2B,C). Furthermore, all BAs showed a restraint effect on the levels of IL-1β in the culture medium (Figure 2D). These results suggest that BA can inhibit the NLRP3 inflammasome and show an anti-inflammatory effect in inflammatory conditions.

### SP1 Mediates the Activation of NLRP3 Inflammasome in Non-Inflammatory Conditions

**FIGURE 3:**
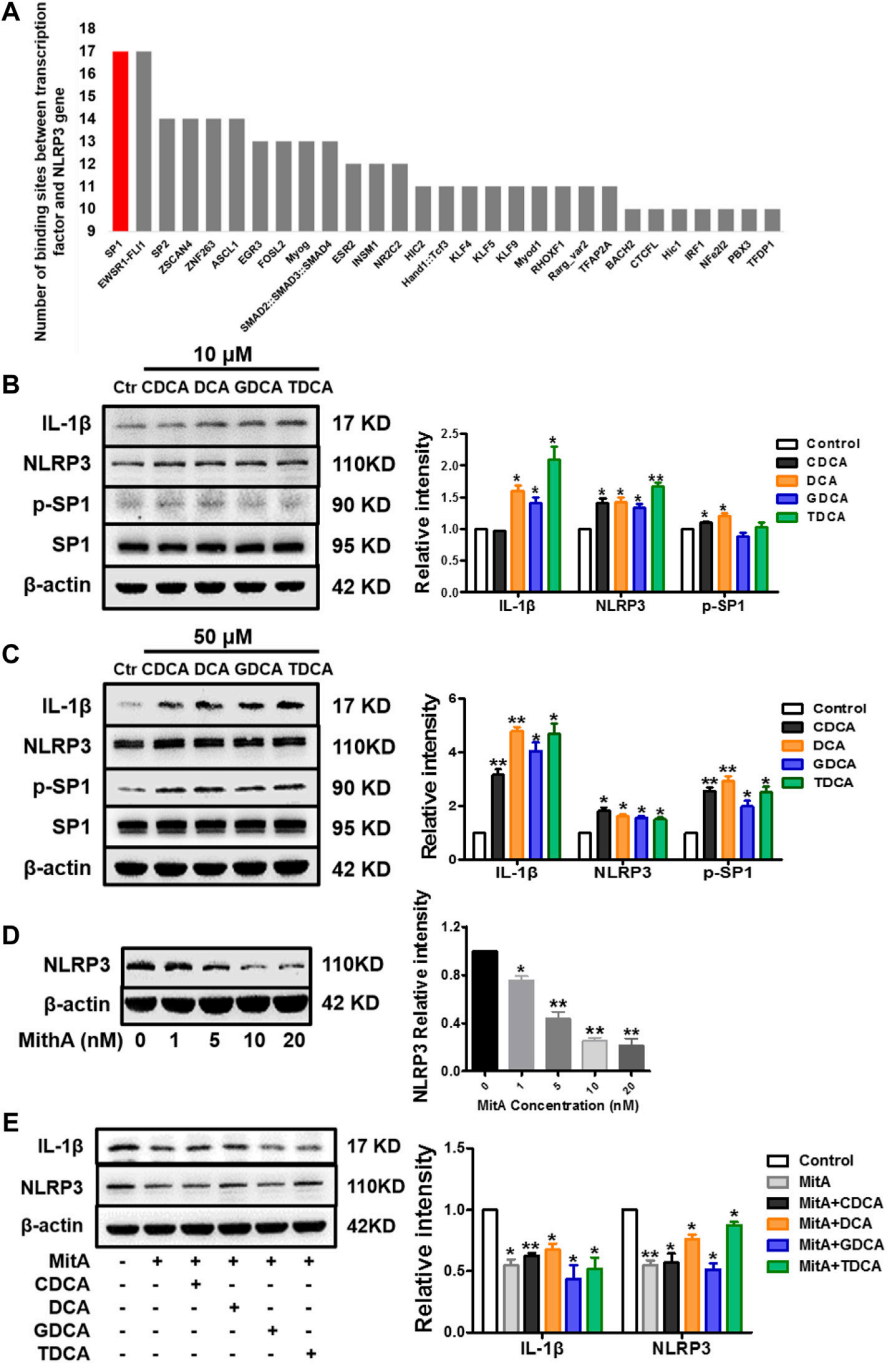
SP1 mediates the activation of NLRP3 inflammasome in non-inflammatory conditions in macrophages. **(A)** Ranking of binding sites between different transcription factors and NLRP3 gene (data were from the eukaryotic promoter database, from −2,000 to 100 bp relative to TSS, and the cut-off was *p* = 0.001) (https://epd.epfl.ch). Western blot analysis of IL-1β, NLRP3, and SP1 in macrophages differentiated from THP-1 monocytes with **(B)** 10 μM and **(C)** 50 μM BA treatments for 4 h. **(D)** NLRP3 expression analyzed by Western blot with different concentrations of MitA for 48 h. **(E)** Western blot analysis of IL-1β and NLRP3 after cells were pretreated with 10 nM of MitA for 48 h prior to the stimulation with BAs at 50 μM for 4 h. Data are presented as mean ± SD (*n* = 3). Statistical analysis was performed using Student’s *t* test (*p < 0.05, **p < 0.01).

SP1 is one of the transcription factors of the NLRP3 gene with the highest score based on the number of binding sites (Figure 3A). Therefore, we investigated whether SP1 is essential in mediating the activation process. Interestingly, we found that p-SP1 was significantly promoted by BA in non-inflammatory conditions, especially at 50 μM, which shared a similar trend with IL-1β and NLRP3. However, there were no obvious changes in SP1 with both 10 and 50 μM of BA (Figures 3B,C). Then, we stimulated macrophages with a series of concentrations of MitA (according to Supplementary Figure S2B), a selective inhibitor of SP1. The results show that MitA (<10 nM) could inhibit NLRP3 in a dose-dependent manner (Figure 3D). Moreover, after 48 h of pretreatment with MitA (Seznec et al., 2011; Liu et al., 2018), the NLRP3 inflammasome activation induced by BA was reversed (Figure 3E), indicating that SP1 mediates the activation of NLRP3 by BA in non-inflammatory conditions. We also determined the levels of SP1 and p-SP1 in inflammatory conditions with BA treatment, and a very mild increase of p-SP1 was observed compared to that in non-inflammatory conditions (Supplementary Figure S3A).

### TGR5 Participates in the Inhibition of NLRP3 Inflammasome Under Inflammatory Conditions

**FIGURE 4:**
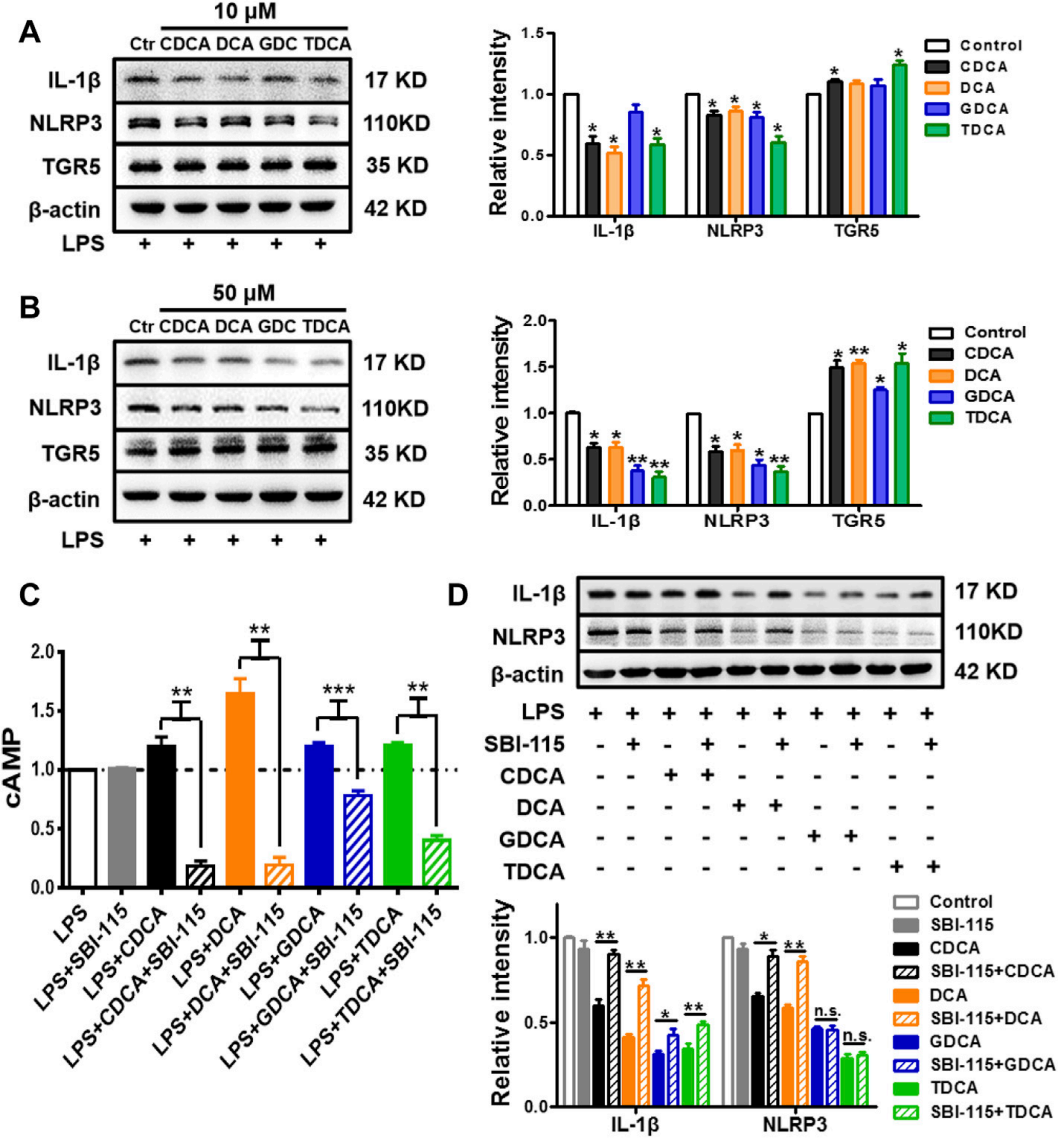
TGR5 participates in the inhibition of NLRP3 inflammasome in inflammatory conditions in macrophages. IL-1β, NLRP3, and TGR5 expressions determined by Western blotting. The cells were pretreated with 250 ng/ml of LPS for 1 h and stimulated with BAs at **(A)** 10 μM and **(B)** 50 μM for 3 h. LPS-pretreated macrophages were treated with 100 μM of SBI-115 and 50 μM of BAs, and the supernatants and lysates were prepared. **(C)** Level of cAMP in the supernatants analyzed by ELISA. **(D)** IL-1β and NLRP3 expression in the lysates. Data are presented as mean ± SD (*n* = 3). Statistical analysis was performed using Student’s *t* test (*p < 0.05, **p < 0.01, and ***p < 0.001).

As previous studies suggested, BA can inhibit the activation of NLRP3 inflammasome via TGR5 signaling (Guo et al., 2016a). In the present study, we confirmed that in inflammatory circumstances induced by 250 ng/ml of LPS, BA promoted the expression of TGR5 and inhibited the expression of IL-1β and NLRP3 at the same time (Figures 4A,B). Then, 250 ng/ml of LPS-pretreated macrophages was simultaneously stimulated with BA and SBI-115, an antagonist of TGR5 (Masyuk et al., 2017) (Supplementary Figure S2C). As shown in Figure 4C, 100 μM of SBI-115 could reverse the increase of the cAMP content in the culture medium caused by BA. It is obvious that among the four BAs, DCA promoted cAMP most remarkably and SBI-115 showed the strongest effect against DCA (Figure 4C) as well, which is consistent with the previous findings that TGR5 is differentially activated by BA in the strength order of DCA > LCA > CDCA > CA (Guo et al., 2016b; Wahlström et al., 2016). Antagonizing TGR5 with SBI-115 offsets the inhibitory effect of BA, especially DCA and CDCA, on IL-1β and NLRP3 to some extent (Figure 4D), indicating that TGR5 is involved in the inhibition of NLRP3 inflammasome by BA in inflammatory conditions. The level of TGR5 was determined as well in non-inflammatory conditions after BA treatment, and the increase is very limited compared to that in inflammatory conditions (Supplementary Figure S3B).

### Ameliorating CPT-11-Induced Colitis Utilizing Inflammatory-Dependent Bidirectional Effects of Bile Acids on NLRP3 Inflammasome

**FIGURE 5:**
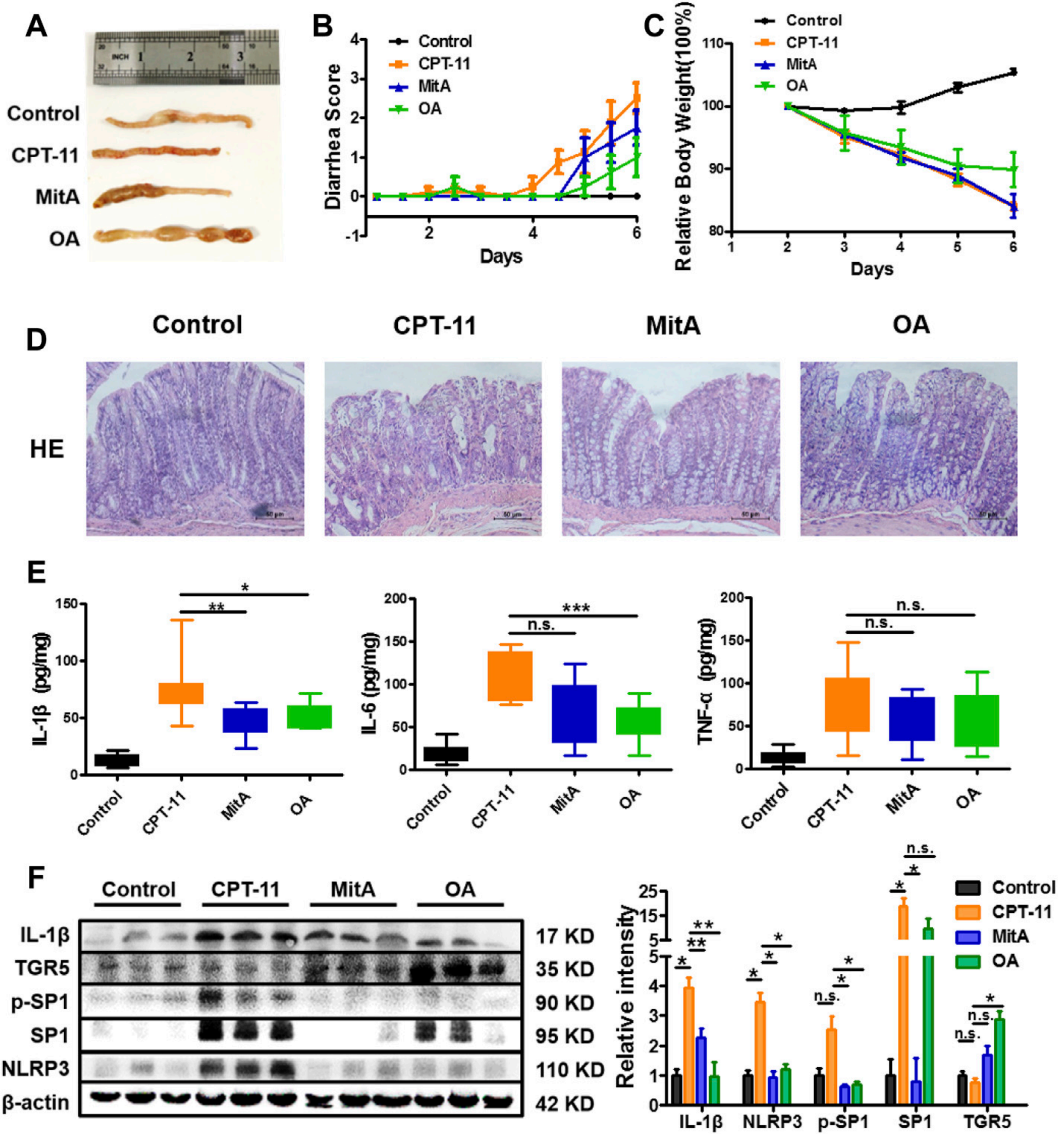
Amelioration of the CPT-11-induced diarrhea in rats. **(A)** Representative images of colon tissues from each group at the last experimental day. **(B)** The diarrhea score of each rat was monitored twice daily and is presented as mean ± SD (*n* = 8). **(C)** The change of the relative body weight of rats in different groups upon CPT-11 administration. **(D)** HE staining was performed on rat colon after drug administration (at ×200 magnification). **(D)** IL-6, IL-1β, and TNF-a levels in rat colons. **(E)** Expression of IL-1β, NLRP3, TGR5, SP1, and phospho-SP1 in rat colons determined by Western blotting. Data are presented as mean ± SD (*n* = 3). Statistical analysis was performed using Student’s *t* test (*p < 0.05, **p < 0.01, ***p < 0.001, and n.s., not significant; the statistical analysis results in panels **(B,C)** can be found in Supplementary Tables S1 and S2).

Based on what has been found *in vitro*, *in vivo* experiments were designed to confirm whether an anti-inflammatory effect can be achieved in CPT-11-induced colitis (Supplementary Figure S1). However, beyond our expectation, the combination of MitA and OA led to a much lower survival rate compared to the MitA or OA group (Supplementary Figure S4), making the statistical comparisons between MitA + OA and other groups difficult. Therefore, only the data of control, model, MitA, and OA groups are presented. Inflammation and hemorrhage in colon and watery stool were observed in the model group. Notably, SP1 inhibitor MitA or TGR5 agonist OA dramatically alleviated these adverse symptoms (Figure 5A). Besides, OA could significantly ameliorate the weight loss and diarrhea induced by CPT-11, while MitA hardly showed capability in this (Figures 5B,C). Histopathological examination showed that MitA and OA improved the colon damage and macrophage infiltration caused by CPT-11 (Figure 5D and Supplementary Figure S5). Moreover, we found that MitA had a stronger effect on IL-1β, while OA showed a more powerful action on IL-6, and both of them had a relatively weaker effect on TNF-α (Figure 5E).

To further clarify whether MitA and OA functioned via inhibiting SP1 or activating TGR5, we detected the expression of SP1, p-SP1, TGR5, IL-1β, and NLRP3 in the rat colons. The results showed that p-SP1 and SP1 were significantly inhibited in the MitA group compared with those in the model group, while TGR5 was significantly promoted in the OA group. Besides, IL-1β and NLPR3 were down-regulated in the groups of MitA and OA (Figure 5F). Taken together, our work demonstrated that SP1 and TGR5 could be promising intervention targets for alleviating chemotherapy-induced intestinal toxicity.

## Discussion

Emerging evidence has shown a strong association between BA and intestinal diseases. Importantly, almost all inflammatory intestinal diseases are accompanied with BA dysregulation (for example, inflammatory bowel disease (Zhou et al., 2014; Fitzpatrick and Jenabzadeh, 2020) and chemotherapy-induced colitis (Muls et al., 2016; Andreyev et al., 2021)). For example, previous studies have revealed the involvement of disturbed BA metabolism in CPT-11-induced colitis (Fang et al., 2016). On the other hand, inflammasomes, such as NLRP3 and AIM2, have been proved to play crucial roles in CPT-11-induced gastrointestinal toxicity (Li et al., 2015; Lian et al., 2017). Moreover, existing studies suggest that repressing NLRP3 inflammasome can ameliorate intestinal inflammatory injury (Gong et al., 2018; Shao et al., 2019; Cao et al., 2020) as well as CPT-11-induced colitis (Li et al., 2015; Huang et al., 2020). Therefore, it is essential to explore the BA–NLRP3 inflammasome axis in the CPT-11 intestinal injury. However, as mentioned before, there are controversial findings regarding the effect of BA on the NLRP3 inflammasome.

In this study, we discovered that BA could activate NLRP3 inflammasome through promoting the transformation of SP1 into p-SP1 under non-inflammatory conditions. SP1 is a transcription factor that is well known for its significant role in cell growth, differentiation, apoptosis, and carcinogenesis (Beishline and Azizkhan-Clifford, 2015; Vizcaíno et al., 2015). Its encoded proteins are involved in many essential cellular processes such as cell differentiation and immune responses (Vellingiri et al., 2020). According to existing studies, multiple post-translational modifications could mediate SP1 activation (Higuchi et al., 2004; González-Rubio et al., 2015) including phosphorylation, O-linked glycosylation, acetylation, SUMOylation, or ubiquitylation (Beishline and Azizkhan-Clifford, 2015), in which BA might be involved. On the other hand, SP1 also correlates to colorectal cancer (Chen et al., 2018; Yu et al., 2018). MitA, the selective inhibitor of SP1, is reported to inhibit colorectal cancer (Quarni et al., 2019; Li et al., 2020). Therefore, we speculate that the combination of MitA or other SP1 inhibitors and CPT-11 might achieve a startling effect of reducing the side effect and enhancing the anticancer efficacy simultaneously in the treatment of colorectal cancer.

In inflammatory conditions, we show in this study that TGR5 participates in the inhibition of NLRP3 inflammasome by BA *in vitro*, while *in vivo* TGR5 was significantly promoted by OA, but not affected by CPT-11 and MitA treatments. TGR5 is a metabolic regulator involved in glucose tolerance, energy expenditure, and inflammation (Holter et al., 2020). As a member of the G-protein-coupled receptor (GPCR) superfamily, TGR5 can be activated by BA and then elevate intracellular cAMP levels (Guo et al., 2016b; Keitel et al., 2019). Recently, there are studies reporting that TGR5 has an inhibitory effect on NLRP3. For instance, BA could lead to the phosphorylation of NLRP3 via the TGR5–cAMP–PKA axis, which serves as a critical brake on the NLRP3 inflammasome activation (Guo et al., 2016a). In another study, BA reduced the nuclear translocation of the nuclear factor (NF)-κB p65 and lowered the NF-κB transcriptional activity to depress NLRP3 inflammasome through the TGR5–cAMP pathway (Keitel and Häussinger, 2018). In addition, TGR5 has crucial protective functions in augmenting bile composition and cytokine release in cholestasis (Deutschmann et al., 2018; Willis et al., 2020). Moreover, TGR5 is proved to improve colitis by modulating the integrity of intestinal barrier and immune response (Cipriani et al., 2011; Biagioli and Carino, 2017; Sorrentino et al., 2020), indicating its potential in alleviating chemotherapy-induced intestinal toxicity as an intervening target.

In this study, we investigated the interaction between BA and TGR5 or SP1 in inflammatory or non-inflammatory conditions *in vitro*, respectively. Studies manifest that DCA is a more potent ligand of TGR5 than CDCA; it is therefore expected that DCA has a more propounding effect on both NLRP3 and IL-1β than CDCA. However, in our study, we observed that CDCA (50 μM) exhibited a stronger inhibitory effect on NLRP3 and IL-1β. In addition, our results showed that although DCA exhibited a more promotive effect on the transformation of SP1 into active p-SP1 than CDCA, it only more strongly promoted IL-1β but not NLRP3. We speculate that the ultimate effect of BA on NLRP3 inflammasome is a converged result from the opposite actions of SP1 and TGR5 and potentially other mediators. Besides, since there is no clear borderline between inflammatory and non-inflammatory conditions, at what point BA switches its role from agonist to antagonist needs to be further explored.

*In vivo*, we proved that MitA and OA are effective against CPT-11-induced colitis. We assumed that the CPT-11-induced colitis is a chronic progress from inflammation initiation to recovery instead of a 100% inflammatory condition. The accumulation of BAs after CPT-11 administration in colon could promote the phosphorylation of SP1 and enhance the inflammation. As the inflammation progresses, BAs could promote the expression of TGR5 and relieve the inflammation reaction to some extent. Our data show that CPT-11 exposure leads to an increased expression of SP1 and p-SP1, while it has no significant effect on the level of TGR5. MitA significantly inhibited SP1 and p-SP1 and ameliorated inflammation, while OA inhibited NLRP3 inflammasome and ameliorated colitis through promoting TGR5. The results indicate that inflammatory and non-inflammatory mechanisms may co-exist in CPT-11-caused colitis.

We also explored whether the combination of MitA and OA was more effective. However, the mortality rate was as high as 70% in the MitA+OA group. MitA is an anticancer antibiotic and has been reported to be effective in various types of cancers, including colorectal cancer, testicular carcinoma, prostate cancer, etc. (Choi and Choi, 2018; Liu et al., 2018; Novakova et al., 2018; Quarni et al., 2019). In our pre-experiments, three different doses of MitA (0.05, 0.15, and 0.25 mg/kg) were administered, and body weight, diarrhea score, pathological changes, and inflammatory factors were recorded or determined. No obvious adverse effect of concern was observed (data not shown). OA belongs to the pentacyclic triterpene family, as a weak agonist of TGR5, and is known for its hepato-protective effect. It has also been reported that OA is effective in relieving dextran sodium sulfate-induced colitis (Kang et al., 2015; Sen, 2020). OA is generally recognized safe in a wide range of dosages to rats; for example, no obvious adverse effect was observed in rats receiving 120 mg/kg of OA for 9 weeks (Pollier and Goossens, 2012; Madlala et al., 2015). In this context, we think there might be two reasons for the unexpected, high mortality of the combination. One is the drug–drug interaction, which can cause changes in the drug concentration in local tissues and alter drug effect or toxicity (Gessner et al., 2019). The other is intervening SP1 and TGR5 at the same time probably over-regulated the NLRP3 inflammasome and caused unknown fatal side effects. Further experiments are needed to explore the actual mechanisms.

In conclusion, we demonstrated that *in vitro* BA could activate NLRP3 inflammasome in non-inflammatory conditions mediated by SP1 and inhibit NLRP3 inflammasome in inflammatory conditions via TGR5. Treating rats receiving CPT-11 with MitA to inhibit SP1 or OA to activate TGR5 can alleviate the colitis. Our findings may shed lights on the discovery of effective interventions for reducing chemotherapy-induced colitis.

## Data Availability

The original contributions presented in the study are included in the article/Supplementary Material, further inquiries can be directed to the corresponding authors.
