# Supplementary material for: Inflammatory-Dependent Bidirectional Effect of Bile Acids on NLRP3 Inflammasome and Its Role in Ameliorating CPT-11-Induced Colitis
Source: Front Pharmacol. 2022 May 31;13:677738. doi: 10.3389/fphar.2022.677738 (PMC9193974; doi:10.3389/fphar.2022.677738)
Supplement: Supplementary file 1 [file DataSheet1.docx]

Supplementary Material

Inflammatory-dependent Bidirectional Effect of Bile Acids on NLRP3 Inflammasome in CPT-11-Induced Colitis

**Chuyao Liao^1^, Di Wang^1^, Siyuan Qin^1^, Ying Zhang^1^, Jie Chen^1^, Bei Tan^1^, Ruijie Xu^1^, Fengguo Xu^1*^, Pei Zhang^1*^**

^1^ Key Laboratory of Drug Quality Control and Pharmacovigilance (Ministry of Education), State Key Laboratory of Natural Medicine, China Pharmaceutical University, Nanjing 210009, P. R. China

*** Correspondence:**Pei Zhang (Tel/Fax: +86-25-83271021; Email: peizhang@cpu.edu.cn)

Fengguo Xu (Tel/Fax: +86-25-83271021; Email: fengguoxu@cpu.edu.cn)


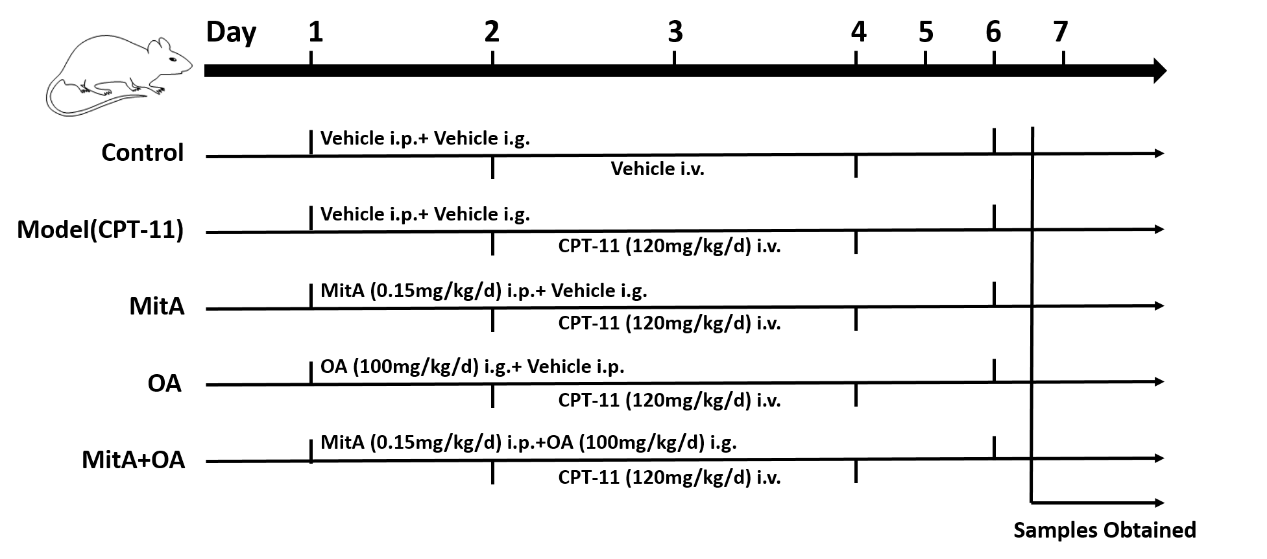


**Figure S1. Procedure of the animal experiment.**


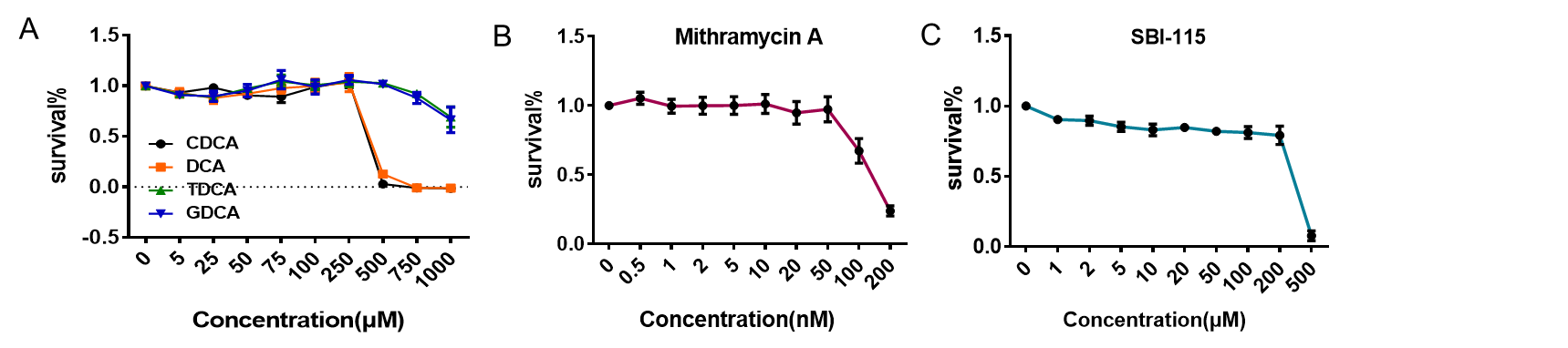


**Figure S2. Cell viability assay.** Macrophages differentiated from THP-1 monocytes were treated with (A) CDCA, DCA, GDCA, and TDCA, (B) Mithramycin A, and (C) SBI-115 for 48 h with indicated concentrations. Cell viability was measured by MTT assay.


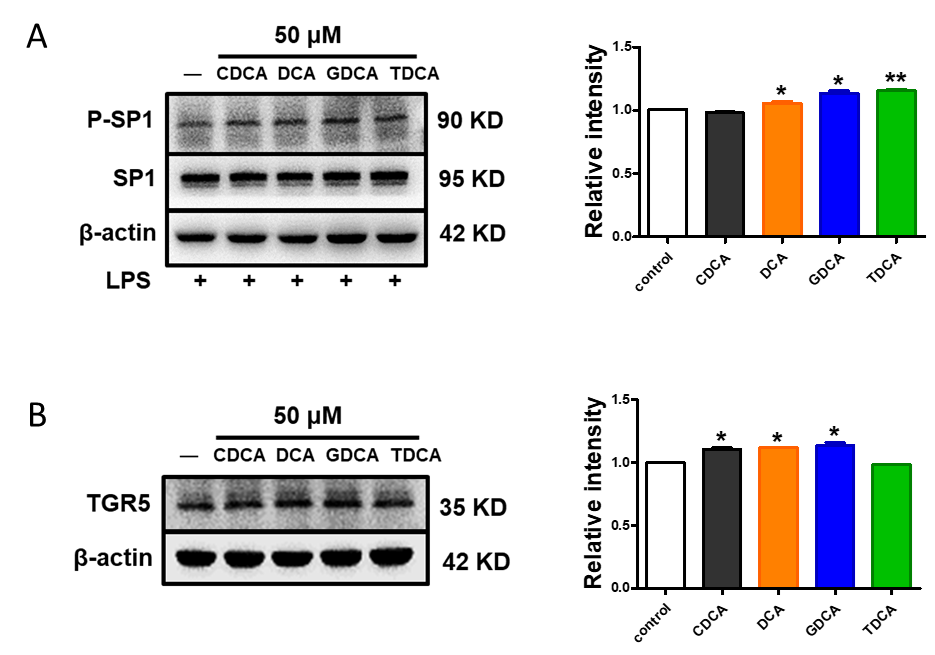


**Figure S3. The expression of p-SP1 in inflammatory condition and TGR5 in non-inflammatory condition**. Western blotting analysis of SP1and TGR5 in macrophages differentiated from THP-1 monocytes with (A) 50 μM BA treatment for 4h, and (B) 50 μM for 3h after 250 ng/mL LPS pretreatment for 1h.


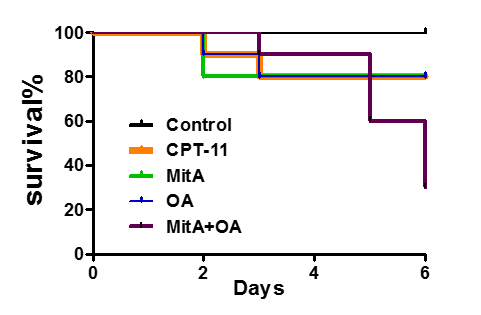


**Figure S4. Rat survival analysis.** Kaplan–Meier survival curves of model group *vs* MitA+OA group through the entire experimental period.


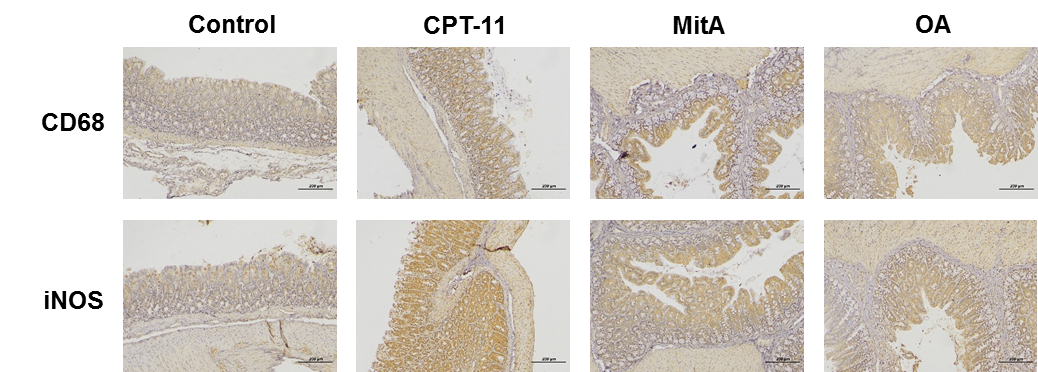


**Figure S5. Immunohistochemistry analysis of rat colon.** Immunohistochemistry analyses of CD68 and iNOS were performed on colon tissue after drug administration (100× magnification).

**Table S1. Statistical analysis of Figure 5B**

| Day | Control group | MitA group | OA group |
| --- | --- | --- | --- |
| 4 | N.S. | N.S. | N.S. |
| 4.5 | * | * | * |
| 5 | N.S. | N.S. | N.S. |
| 5.5 | * | N.S. | N.S. |
| 6 | *** | N.S. | * |

Note: Compared with CPT-11 group, ^*^P < 0.05, ^**^P < 0.01, ^***^P < 0.001, N.S., not significant.

**Table S2. Statistical analysis of Figure 5C**

| Day | Control group | MitA group | OA group |
| --- | --- | --- | --- |
| 3 | * | N.S. | N.S. |
| 4 | ** | N.S. | N.S. |
| 5 | *** | N.S. | N.S. |
| 6 | *** | N.S. | * |

Note: Compared with CPT-11 group, *P < 0.05, **P < 0.01, ***P < 0.001, N.S., not significant.
